# IL-4 Receptor-Alpha-Dependent Control of *Cryptococcus neoformans* in the Early Phase of Pulmonary Infection

**DOI:** 10.1371/journal.pone.0087341

**Published:** 2014-01-27

**Authors:** Andreas Grahnert, Tina Richter, Daniel Piehler, Maria Eschke, Bianca Schulze, Uwe Müller, Martina Protschka, Gabriele Köhler, Robert Sabat, Frank Brombacher, Gottfried Alber

**Affiliations:** 1 Institute of Immunology/Molecular Pathogenesis, Center for Biotechnology and Biomedicine, College of Veterinary Medicine, University of Leipzig, Leipzig, Germany; 2 Gerhard-Domagk-Institute of Pathology, University of Münster, Münster, Germany; 3 Interdisciplinary Group of Molecular Immunopathology, Dermatology/Medical Immunology, University Hospital Charité, Berlin, Germany; 4 International Center for Genetic Engineering and Biotechnology (ICGEB), Cape Town & Institute of Infectious Diseases and Molecular Medicine (IIDMM), University of Cape Town, Cape Town, South Africa; University of Minnesota, United States of America

## Abstract

*Cryptococcus neoformans* is an opportunistic fungal pathogen that causes lung inflammation and meningoencephalitis in immunocompromised people. Previously we showed that mice succumb to intranasal infection by induction of pulmonary interleukin (IL)-4Rα–dependent type 2 immune responses, whereas IL-12-dependent type 1 responses confer resistance. In the experiments presented here, IL-4Rα^−/−^ mice unexpectedly show decreased fungal control early upon infection with *C. neoformans*, whereas wild-type mice are able to control fungal growth accompanied by enhanced macrophage and dendritic cell recruitment to the site of infection. Lower pulmonary recruitment of macrophages and dendritic cells in IL-4Rα^−/−^ mice is associated with reduced pulmonary expression of CCL2 and CCL20 chemokines. Moreover, IFN-γ and nitric oxide production are diminished in IL-4Rα^−/−^ mice compared to wild-type mice. To directly study the potential mechanism(s) responsible for reduced production of IFN-γ, conventional dendritic cells were stimulated with *C. neoformans* in the presence of IL-4 which results in increased IL-12 production and reduced IL-10 production. Together, a beneficial role of early IL-4Rα signaling is demonstrated in pulmonary cryptococcosis, which contrasts with the well-known IL-4Rα-mediated detrimental effects in the late phase.

## Introduction

*Cryptococcus neoformans* is an opportunistic, facultative intracellular basidiomycete acquired by inhaling spores or desiccated fungi. *C. neoformans* has the potential to cause life-threatening meningoencephalitis in immunocompromised individuals such as organ transplant recipients or HIV-infected patients [1]–[3]. In fact, HIV-related cryptococcal meningitis is responsible for more than half a million death cases per year in sub-Saharan Africa and represents the fourth most common cause of death after malaria, diarrheal diseases, and childhood-cluster diseases excluding HIV [4]. Moreover, *C. neoformans* can cause an allergic bronchopulmonary mycosis characterized by production of Th2 cytokines (e.g. interleukin (IL)-4, IL-5, and IL-13), elevated levels of serum IgE, recruitment of eosinophils, and alternative activation of macrophages [5]–[8]. Together with mucus hyperproduction by bronchial epithelial cells all of these features are characteristic for allergic asthma, and lead to smooth muscle hyperreactivity and chronic airway obstruction. The differentiation of Th2 cells plays an important role in asthma and Th2 cytokines, especially IL-4 and IL-13 which both can bind to the IL-4 receptor-alpha chain [9] (IL-4Rα) and exacerbate disease [10], [11]. Finally, mice succumb to *C. neoformans* infection if no protective Th1 polarization is induced [12]–[15]. In contrast, depending on the mouse strain used, the route of infection, the size of the inoculum, and the strain of *C. neoformans* IL-4 deficiency was found to lead either to increased or reduced survival times [5], [14], [16], [17]. For some other infection models, including a fungal pathogen (e.g. *Candida albicans*), it was shown, that IL-4 can be involved in the induction of Th1 immune responses and elevated IFN-γ production [18]–[20]. Due to the protective *in vivo* effect researchers turned their focus on the target cells for IL-4 and it has been shown that in human mononuclear cells as well as in human and mouse dendritic cells IL-4 exerts a positive effect on the production of bioactive IL-12 most likely by inhibiting IL-10 expression [21]–[23].

IL-4 can mediate its effects by binding to two different types of heterodimeric IL-4 receptors designated as the type I and the type II IL-4R. Both types share the IL-4Rα chain and are able to respond to IL-4 as it binds to the IL-4Rα chain with high affinity [9]. To form the type I receptor, the IL-4Rα chain interacts with the common γ chain. After cloning and characterization of the low affinity IL-13Rα1 and the high affinity IL-13Rα2 chain it became evident that the IL-4Rα chain is also part of the IL-13 receptor [24]–[26]. Binding of IL-13 is restricted to IL-4R type II, whereas IL-4 can bind both receptor types. The common γ chain expression is restricted to hematopoietic cells. Therefore, type I IL-4R is mainly expressed in hematopoietic cells, whereas type II IL-4R is ubiquitously expressed [27].

In the experiments described here, we analyzed the impact of IL-4Rα expression on the early immune responses in a chronic pulmonary cryptococcosis model. We show that, in contrast to the late Th2-driven phase of infection, within the first two weeks of infection IL-4Rα signaling is able to elicit potent macrophage and dendritic cell recruitment and elevated production of IFN-γ and nitric oxide associated with better fungal growth control. This beneficial role of early IL-4Rα function is intriguing as wild-type (WT) mice that are protected in the initial phase of infection show features of an otherwise type 2-biased immune response.

## Materials and Methods

### Ethics statement

All mouse experiments were performed according to protocols (Permit number: 24-9168.11/14/19) approved by the Animal Care and Usage Committee of the Landesdirektion Sachsen. All efforts were made to minimize suffering.

### Mice

For all experiments female mice on C57BL/6J background were used. Age-matched (8 to 14 weeks) wild-type (WT) mice (Janvier, Le Genest Saint Isle, France) and IL-4Rα deficient mice (IL-4Rα^−/−^) [28], backcrossed onto C57BL/6J background for 9 generations, were kept under specific pathogen-free conditions in accordance with the guidelines approved by the Animal Care and Usage Committee of the Landesdirektion Sachsen. The mice were tested periodically for pathogens, in accordance with the recommendations for health monitoring of mice provided by the Federation of European Laboratory Animal Science Associations accreditation board. No pinworms and other endo- and ectoparasites were detectable. Sterile water and food were given *ad libitum*.

### Infection of mice with *C. neoformans*

Encapsulated *C. neoformans*, strain 1841, serotype D, originally obtained from F. Hoffmann-La Roche Ltd, Basel, Switzerland [14], was kept as a frozen stock in skim milk and was grown in Sabouraud dextrose medium (2% glucose and 1% peptone; Sigma, Deisenhofen, Germany) overnight on a shaker at 30°C. Cells were washed twice in sterile phosphate-buffered saline (PBS), resuspended in PBS, and counted in a hematocytometer. Inocula were diluted in PBS to a concentration of 2.5×10^4^/mL for intranasal (*i.n.*) infection. Mice were infected by *i.n.* application of 20 µL volumes containing 500 colony forming units (CFUs). Before infection, mice were anesthetized intraperitoneally with a 1:1 mixture of 10% (w/v) ketamine and 2% (w/v) xylazine (Ceva Tiergesundheit, Düsseldorf, Germany).

### Isolation of pulmonary leukocytes and determination of fungal lung organ burden

The preparation of a single cell suspension from lung tissue and isolation of leukocytes was described elsewhere [29]. Briefly, at the time points indicated infected mice were anesthetized with CO_2_, sacrificed by exsanguination, and the circulation was perfused with sterile 0.9% (w/v) sodium chloride solution (Baxter, Unterschleiβheim, Germany). Lungs (left lobe, cranial and caudal right lobe) were removed aseptically, minced with scalpel blades and digested for 30 min at 37°C in RPMI1640 supplemented with 1 mM sodium pyruvate (AppliChem, Darmstadt, Germany), Collagenase D (0.7 mg/ml; Roche Diagnostics, Mannheim, Germany) and DNase IV (30 µg/ml; Sigma Aldrich, Taufkirchen, Germany). After passage through a 100 µm cell strainer (BD Biosciences, Heidelberg, Germany) single cells were resuspended in 1 ml PBS containing 3% (v/v) heat-inactivated fetal calf serum (FCS) (Life Technologies, Darmstadt, Germany) and serial dilutions of aliquots were plated on Sabouraud dextrose agar plates for lung organ burden determination. The plates were incubated for 72 h at 30°C and grown colonies were counted. Following red blood cell lysis and washing with PBS containing 3% (v/v) FCS remaining cells were resuspended in 70% (v/v) Percoll (GE Healthcare Europe GmbH, Freiburg, Germany) and layered under 30% (v/v) Percoll. After density gradient centrifugation cells were removed from the interphase, washed with Iscove's Modified Dulbecco's Medium (IMDM) (GE Healthcare Europe) supplemented with 10% (v/v) FCS, 100 U/ml penicillin, and 100 µg/ml streptomycin, counted using a hematocytometer and used for flow cytometric analyses. For *ex vivo* stimulation, cells were pooled from 3–4 animals, adjusted to 1×10^7^/ml and stimulated with PMA (40 ng/ml; Enzo Life Sciences, Lörrach, Germany) and ionomycin (1 µg/ml; Sigma Aldrich).

### Monoclonal antibodies

Unless otherwise described antibodies labeled with different fluorochromes were from BD Biosciences, eBioscience (Frankfurt, Germany), and BioLegend (Fell, Germany). Following clones were used: anti-CD4 (clone RM4–5), anti-CD11c (clone N418), anti-CD45 (clone 30-F11), anti-Siglec-F (clone E50-2440), and anti-T1/ST2 (clone DJ8, MDbiosciences, Egg, Switzerland). Isotype-matched control antibodies, rat IgG2a (clone eBR2a), Armenian hamster IgG (clone HTK888), rat IgG2b (clone RTK4530), and rat IgG1 (clone eBRG1) were used in all experiments. To detect dead cells and to block unspecific antibody binding cells were incubated with the LIVE/DEAD^®^ Fixable Aqua Dead Cell Stain Reagent (Life Technologies) and rat-anti-mouse CD16/32 (BioLegend, clone 93) prior to incubation with fluorochrome-labeled antibodies.

### Analysis of pulmonary leukocytes by flow cytometry

Purified lung single cells were stained with antibodies described above in different combinations. Briefly, forward scatter vs. dead cells was used to identify living cells. After gating on CD45^+^ leukocytes expression of Siglec-F vs. CD11c was used to identify eosinophils (Siglec-F^+^, CD11c^neg/dim^), alveolar macrophages (Siglec-F^+^, CD11c^+^), and dendritic cells (Siglec-F^neg^, CD11c^+^) [30]. The frequency of T1/ST2^+^ cells was determined after gating on living CD4 expressing cells.

Cells were acquired on a BD FACSCanto II, BD FACS LSRII, and BD LSRFortessa (BD Biosciences) and data were analyzed using FlowJo 7.6.5 (Treestar Inc., Ashland, OR, USA) software.

### Analysis of mRNA expression in lung tissue

From lungs prepared as described above the accessory lung lobe and the lower part of the middle right lung lobe were snap frozen in liquid nitrogen and stored at −80°C until mRNA isolation. Afterwards, snap frozen samples were homogenized in Invisorb® lysing solution (Invitek, Berlin, Germany) during thawing by means of Ultraturrax tissue homogenizer (Jahnke and Kunkel, Staufen, Germany) and treated with 4 mg/ml proteinase K for 1 h (Clontech Laboratories). Isolation of total cellular RNA was done by use of Invisorb® RNA kit II (Invitek). Messenger RNA was reverse transcribed and analyzed in triplicate assays by TaqMan PCR using the ABI Prism 7700 Sequence Detection System (Applied Biosystems, Weiterstadt, Germany) as described previously [31], [32]. The appropriate assays including double-fluorescent probes in combination with assay for the murine house-keeping gene hypoxanthine phosphoribosyl-transferase 1 (HPRT) were developed by ourselves (HPRT and IFN-γ) or purchased from Applied Biosystems (CCL2, CCL20). The following primers and probes were used: HPRT for: 5′-ATCATTATGCCGAGGATTTGGAA-3′, rev: 5′-TTGAGCACACAGAGGGCCA-3′, probe: 5′-TGGACAGGACTGAAAGACTTGCTCGAGATG-3′; IFN-γ for: 5′-CAACAGCAAGGCGAAAAAGG-3′, rev: 5′-AGCTCATTGAATGCTTGGCG-3′, probe: 5′-TGCATTCATGAGTATTGCCAAGTTTGAGGTC-3′. Expression levels were calculated relative to the data for HPRT obtained with the every matching assay.

### Histopathological analysis

To evaluate the pulmonary inflammation, distribution of cryptococci and mucus production by bronchial epithelial cells the upper part of the middle right lung lobe was fixed in 4% neutral-buffered formaldehyde (Carl Roth GmbH, Karlsruhe, Germany) and embedded in paraffin. Sections were stained with H&E for the detection of eosinophils and other leukocytes or with periodic acid Schiff reagent to visualize mucus production by bronchial epithelial cells and distribution of cryptococci. The percentage of PAS^+^ brochial epithelial cells was determined by an independent investigator by counting PAS^+^ and PAS^−^ bronchial epithelial cells of 5 cross-sections of proximal bronchi of two slices per lung (different lung regions), with a total of 10 cross sections per mouse [33].

### Generation and stimulation of conventional dendritic cells

Bone marrow derived dendritic cells (BMDC) were generated as described earlier [34]. Briefly, femur and tibia of C57BL/6J mice were removed and the bone marrow was flushed out with PBS containing 5% (v/v) FCS. Conventional dendritic cells were generated by cultivation of bone marrow cells (2×10^5^/ml) for 8 days in RPMI 1640 supplemented with 10% (v/v) FCS, 100 U/ml penicillin, 100 µg/ml streptomycin, 1% (v/v) essential and non-essential amino acids (GE Healthcare Europe) 1 mM sodium pyruvate, 50 µM β-mercaptoethanol (Sigma-Aldrich) and 10% (v/v) GM-CSF containing supernatant at 37°C in a humidified atmosphere containing 5% (v/v) CO_2_. After harvesting, the cells were adjusted to 5×10^5^/ml and stimulated with *C. neoformans*, strain 1841 in the presence or absence of 25 U/ml recombinant IL-4 (Peprotech, Hamburg, Germany). For control, cells incubated with medium alone were used.

### Detection of cytokines and nitric oxide in cell culture supernatants

Cytokines in cell culture supernatants were measured by sandwich ELISA. For the measurement of IFN-γ the rat IgG1 monoclonal antibody AN18 was used as a capture antibody. Detection was performed using a rat IgG1 monoclonal antibody XMG1.2 labeled with horseradish peroxidase (both antibodies were provided by F. Hoffmann-La Roche Ltd, Basel, Switzerland). IL-12p70 was analyzed by coating ELISA plates with monoclonal antibody 2B5 and detection was done with biotinylated goat-anti-mouse IL-12p40 IgG (provided by M. Gately, F. Hoffmann-La Roche Ltd, Nutley, NJ, U.S.A.). IL-10 was detected by using the murine IL-10 development kit (Peprotech) according to manufacturer instructions.

Biotinylated antibodies were visualized by incubation with horseradish peroxidase labeled streptavidin (Southern Biotechnology Associates, Birmingham, AL, U.S.A.) and the TMB Microwell Peroxidase Substrate System (Gaithersburg, MD, U.S.A.). The reaction was stopped by the addition of 1M H_3_PO_4_ and optical densities were determined at 450 nm (reference 630 nm) using a Spectra-max 340 ELISA reader (Molecular Devices, Munich, Germany).

To analyze the production of nitric oxide a colorimetric reaction (Griess reaction) was used. The supernatants from density gradient purified and pooled lung leukocytes (3–4 animals per group) stimulated with PMA/ionomycin were incubated with equal amounts of a freshly prepared 1:1 mixture (Griess reagent) of 1% (w/v) Sulfanilamide (Sigma) in 5% (w/v) H_3_PO_4_ and 0.1% (w/v) N-(1-Naphthyl)ethylenediamide (Sigma). Following incubation for 10 min in the dark plates were read at 550 nm (reference 690 nm) with the ELISA reader described above. For the calibration curve ranging from 200 µM to 3.125 µM sodium nitrite was used as standard.

## Results

### Lack of IL-4Rα chain results in increased early fungal organ burden but late resistance against *C. neoformans*

**Figure 1:**
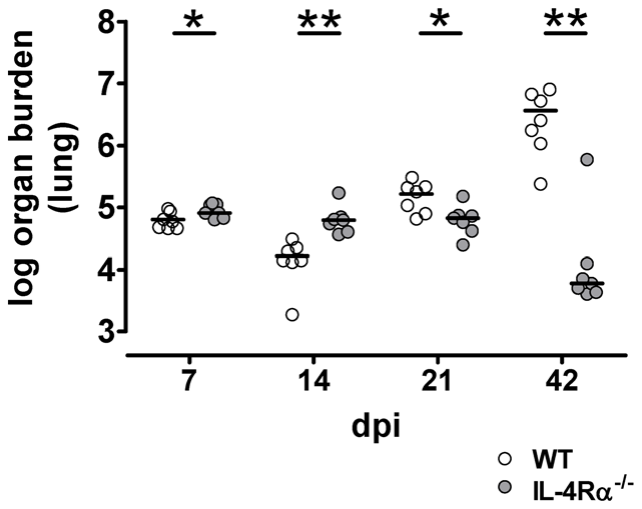
Early fungal growth control in pulmonary infection with *C. neoformans* in the presence of IL-4Rα signaling. Wild-type (WT, open circle) and IL-4Rα^−/−^ (gray circle) mice on C57BL/6J background were infected intranasally with *C. neoformans*. Analysis of fungal burdens in the lung was done at different days *post infectionem* (dpi) as indicated. Shown is data from n = 7 mice per group from one representative of three independent experiments (14 dpi) or from two independent experiments (7; 21 and 42 dpi). Statistical analysis was done using the unpaired Student's t-test (7 dpi) or Mann-Whitney test. *P<0.05; **P<0.01.

Infection with *Cryptococcus neoformans* mainly occurs by inhaling dust particles (e.g. soil, pigeon excreta) contaminated with cryptococcal spores or desiccated cryptococci [35]. Therefore, we used a well-established chronic intranasal infection model to mimic the natural course and route of infection [29]. To analyze the role of the IL-4Rα during cryptococcosis in the initial period of infection we inoculated WT and IL-4Rα^−/−^ mice with 500 colony forming units (CFU) of the highly virulent *C. neoformans* strain 1841 (isolated from a HIV/AIDS patient with cryptococcal meningitis [14]) and analyzed the animals starting at 7 days *post infectionem* (dpi). Surprisingly, mice deficient in IL-4Rα expression show significantly higher lung burdens at early time points after infection (i.e. 7 and 14 dpi) but not at later time points (i.e. 21 and 42 dpi) (Figure 1), when the IL-4Rα^−/−^ mice exhibit significantly lower fungal burdens in the lung. At 70 dpi WT mice continued to show significantly higher lung load than IL-4Rα^−/−^ mice (data not shown), as previously published [36]. These data reveal that, for the first two weeks of infection, control of pathogen load in the lung depends on IL-4Rα expression. Reduction of early fungal growth control in the absence of the type 2 response-associated IL-4Rα signaling was unexpected. Therefore, we focused our further analysis on early time points of the infection (i.e. 7 and 14 dpi).

### IL-4Rα^−/−^ mice show a defect in recruitment of leukocytes to the lung within the first two weeks of infection

**Figure 2:**
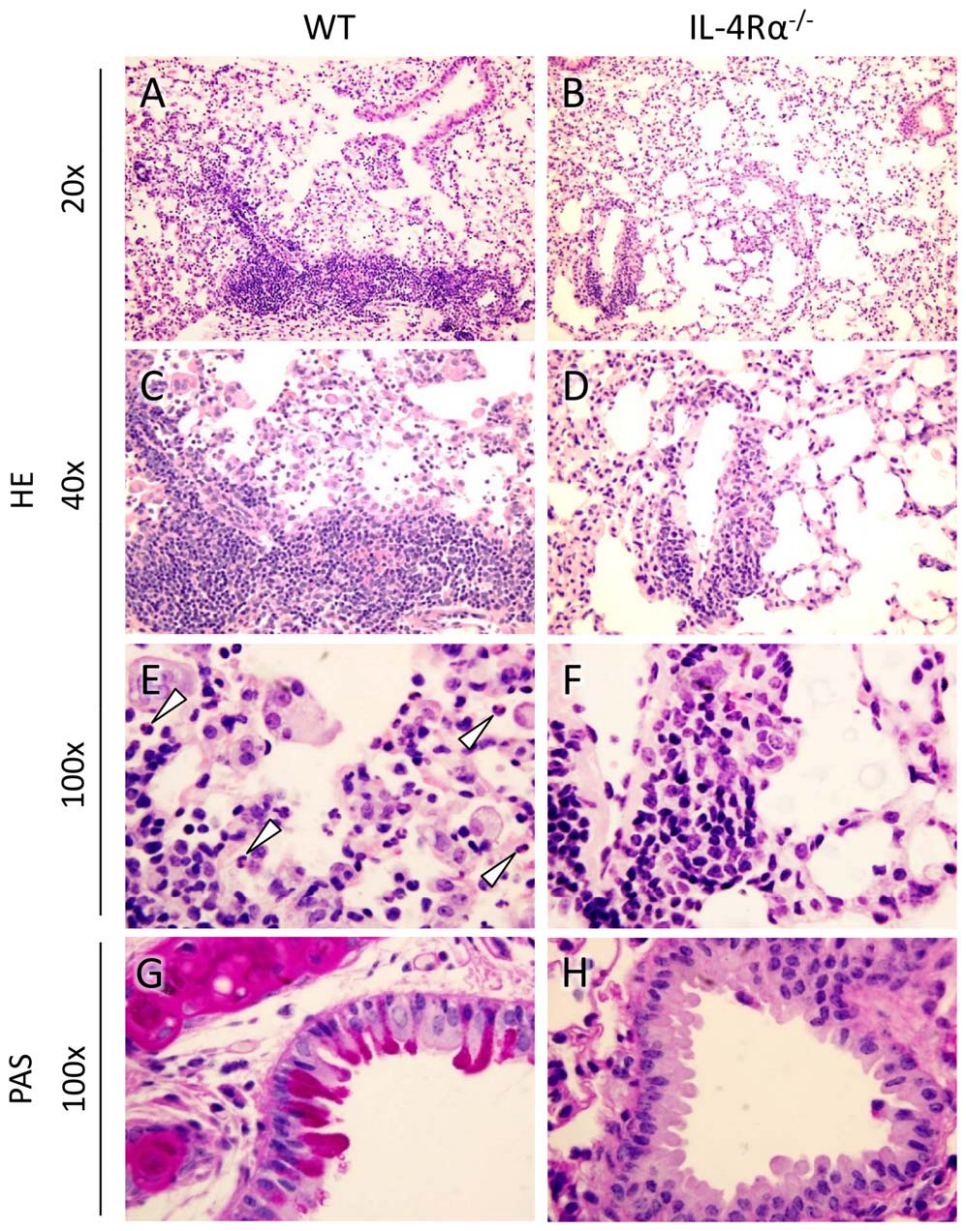
Stronger pulmonary inflammation, eosinophilia, and mucus production in WT as compared with IL-4Rα^−/−^ mice. Lung slices from WT and IL-4Rα^−/−^ mice infected for 14 days were stained with H&E (A-F) and periodic acid Schiff reagent (G, H). Leukocyte infiltration and fungal load are depicted in panels A, C and B, D. Sites of inflammation contain eosinophils (arrowheads) and large, multinucleated macrophages (E) or lymphocytes (F). Mucus production by bronchial epithelial cells is depicted in G and H. One representative experiment out of three with n = 6–7 animals per group is shown.

**Figure 3:**
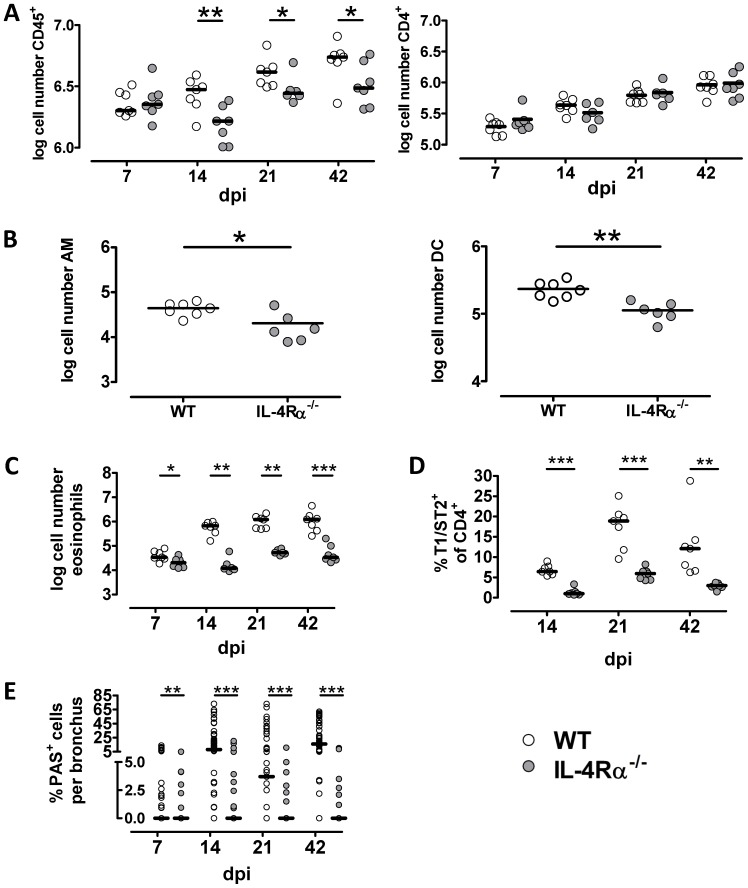
Enhanced alveolar macrophage and dendritic cell infiltration in the presence of IL-4Rα signaling. Lung leukocytes were isolated from WT (open circle) and IL-4Rα^−/−^ (gray circle) C57BL/6J mice infected intranasally with *C. neoformans* at the time points indicated (A, C-E) or at 14 days after infection (B). After perfusion with PBS, density gradient purified cells analyzed by flow cytometry and total numbers of leukocytes (A, left panel), CD4^+^ Th cells (A, right panel), CD11c^+^ Siglec-F^+^ alveolar macrophages (AM, panel B), and CD11c^+^, Siglec-F^−^ dendritic cells (DC, panel B) are shown. Each time point depicts data from n = 6–7 mice of up to two independent experiments. Statistical analysis was done using the unpaired Student's t-test or Mann-Whitney test (21 dpi). *P<0.05; **P<0.01 One representative out of two independent experiments is shown (n = 6–7 mice per group) (A, B). Density gradient purified cells were analyzed by flow cytometry and total numbers of CD11c^neg/dim^, Siglec-F^+^ eosinophils (C) and frequency of T1/ST2^+^ CD4^+^ T helper cells (D) are calculated. Data show one representative experiment out of two (n = 6–7 animal per genotype). Statistical analysis was done using the unpaired Student's t-test or Mann-Whitney test. *P<0.05; **P<0.01; ***P<0.001 Lung sections were stained with periodic acid Schiff reagent and the number of PAS^+^ cells per bronchus was determined from 4–10 bronchi per animal (n = 6–7) (E). Each time point shows data from n = 6–7 mice from one representative of up to three independent experiments. Statistical analysis was performed using the Mann-Whitney test. **P<0.01; ***P<0.001.

In order to characterize the cellular response to the fungus at the site of infection we analyzed the pulmonary inflammatory infiltrates *in situ* by histopathological analysis. 14 days after infection, stronger pulmonary infiltration of leukocytes is visible in WT mice (Figure 2A and C) as compared with IL-4Rα^−/−^ mice (Figure 2B and D). Despite the significantly higher fungal burdens in the lungs of IL-4Rα^−/−^ mice (Figure 1) fewer infiltrating pulmonary leukocytes are observed than in WT mice, consisting mainly of lymphocytes (Figure 2F). In WT mice not only lymphocyte-rich foci are observed, but also more macrophages (with some multinucleated macrophages) participated in the pulmonary inflammatory response (Figure 2E). This is consistent with the quantitative difference in total numbers of alveolar macrophages found by flow cytometry-analysis, described below (see Figure 3B). WT mice, but not IL-4Rα^−/−^ mice, develop a pronounced eosinophilia (Figure 2E and F). Similarly, bronchial mucus production was found in WT but not IL-4Rα^−/−^ mice (Figure 2G and H).

### IL-4Rα^−/−^ mice show a defect in macrophage and dendritic cell recruitment to the lung

To characterize and quantify the pulmonary inflammatory response in more detail in infected WT and IL-4Rα^−/−^ mice we used flow cytometry for analysis of lung leukocytes. As shown in Figure 3A we observed an increasing number of pulmonary leukocytes (i.e. CD45^+^ cells) in WT mice over the time. This increase is much less pronounced in the IL-4Rα^−/−^ group even at 14 dpi when lung fungal burdens in IL-4Rα^−/−^ mice are significantly higher than in WT mice (Figure 1). Recently we demonstrated that IL-4Rα expression on Th cells and macrophages plays a key role in the course of pulmonary cryptococcosis [37], [38]. When analyzing these leukocyte subpopulations, we observed a comparable increase in the number of Th cells in WT and IL-4Rα^−/−^ mice (Figure 3A), whereas the number of alveolar macrophages and dendritic cells was significantly higher in WT as compared with IL-4Rα^−/−^ mice at 14 days after infection (Figure 3B).

In the murine pulmonary model of infection with *C. neoformans*, WT mice show a pronounced type 2 immune response that leads to susceptibility against the fungus [16], [17], [39]. In order to study whether the apparent early resistance of WT mice is associated with early Th2-like immune response parameters, we analyzed the lung leukocytes of infected mice by flow cytometry in more detail. As mentioned before and depicted in Figure 2E the number of eosinophils is elevated in the WT group at each time point analyzed and hence even in the early phase of infection (Figure 3C). Eosinophils account for approximately half of the difference in total leukocyte cell numbers observed between the two groups after infection (Figure 3A). This is noteworthy as eosinophilia is linked to susceptibility [39], [40]. Recently we could show at a late time point of infection with *C. neoformans* that the Th2 cell marker T1/ST2 is associated with enhanced Th2 cell activation and polyfunctionality, ultimately resulting in defective pulmonary fungal control [33]. Interestingly, as early as 14 days after infection, significantly higher frequencies of T1/ST2^+^ Th2 cells are found in WT mice (Figure 3D). Additionally, when counting periodic acid Schiff staining in lung tissue sections we observed prominent mucus production in the WT group, indicated by PAS^+^ epithelial cells (Figure 3E). Taken together, typical features of Th2-related susceptibility can be found early in cryptococcal infection and accompany the early beneficial activity of IL-4Rα signaling. Despite the expected IL-4Rα-dependent type 2 phenotype (i.e. eosinophilia, mucus production, and development of T1/ST2^+^ Th2 cells) WT mice unexpectedly more efficiently control the early fungal growth. This raises the question of additional simultaneously operating type 1 response mechanisms such as IFN-γ-dependent chemokine regulation leading to enhanced phagocytic influx and elevated NO production early during infection.

### Reduced macrophage attracting chemokine and IFN-γ expression with compromised production of nitric oxide in lung tissue of IL-4Rα^−/−^ mice

**Figure 4:**
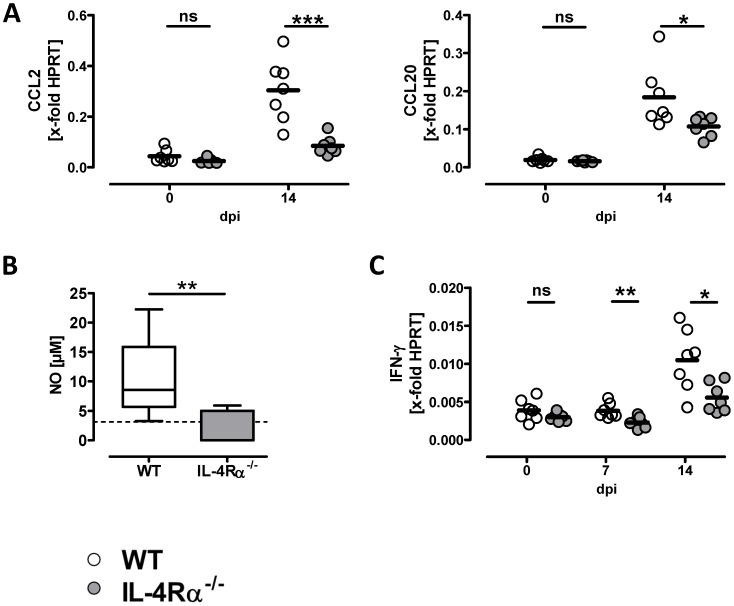
In the presence of IL-4Rα elevated pulmonary chemokine expression, IFN-γ mRNA expression and NO production. Following infection, WT (open circle) and IL-4Rα^−/−^ (gray circle) mice were sacrificed at the time points indicated (A, C) or at 14 days after infection (B). RT-qPCR analyses were done to determine the expression of mRNAs as indicated. Data are derived from one (A, C, 0 and 7 dpi) or one representative out of two (A, 14 dpi) or three (C, 14 dpi) experiments (n = 6–7 mice per genotype and experiment). Statistical analysis was done using the unpaired Student's t-test (ns, not significant; *P<0.05; **P<0.01; ***P<0.001 (A, C)). The concentration of nitric oxide (NO) in cell culture supernatants was determined using the Griess reaction. Pooled data from two different experiments are shown. Dotted line represents detection limit. Statistical analysis was done using the Mann-Whitney test. **P<0.01 (B).

Figure 2E and 3B show an increased influx of macrophages and dendritic cells to the lungs of infected WT mice. We hypothesized that the underlying mechanism of this cellular infiltration is due to differences in expression of chemokines between the two groups. To examine chemokines involved in the attraction of monocytes/macrophages and dendritic cells we studied mRNA expression of CCL2 (monocyte chemoattractant protein-1, MCP-1) and CCL20 (macrophage inflammatory protein-3α, MIP-3α) in lung tissue [41], [42]. Indeed, pulmonary CCL2 and CCL20 expression is significantly higher in WT than IL-4Rα^−/−^ mice infected for 14 days. Naïve WT mice show similar expression of CCL2 and CCL20 as compared with naïve IL-4Rα^−/−^ mice, and for both naïve genotypes the levels are considerably lower than upon infection with *C. neoformans* (Figure 4A).

Concomitantly, reduced production of nitric oxide (NO) in the supernatants of lung leukocytes stimulated *in vitro* using PMA/ionomycin (Figure 4B) is detectable in the IL-4Rα^−/−^ group. Antigen-specific stimulation of pulmonary leukocytes using heat-inactivated acapsular *C. neoformans* strain CAP67 [29] showed a similar difference in NO production between WT and IL-4Rα^−/−^ mice (data not shown). Higher NO production in WT mice raises the question for the underlying regulatory mechanism. Among the three mammalian isoforms of NO synthase (NOS), that catalyze the formation of NO, expression of inducible (i)NOS in turn is stimulated by IFN-γ [43]. The difference in NO production prompted us to analyze the lung tissue samples from mice of the experiments depicted above (Figure 1) for expression of IFN-γ. Using reverse transcription quantitative real-time PCR (RT-qPCR) analysis we found that at 7 and 14 dpi IFN-γ mRNA expression is reduced in lung tissue of infected IL-4Rα^−/−^ mice (Figure 4C). Consistent with the reduced transcription of IFN-γ in the absence of IL-4Rα signaling, lower levels of this cytokine are detectable upon *ex vivo* antigen-specific re-stimulation of lung leukocytes at early (i.e. 7 dpi) but not late (i.e. 42 dpi) time points of infection (data not shown). No difference in pulmonary IFN-γ expression between the two groups is detectable in naïve mice (Figure 4C).

**Figure 5:**
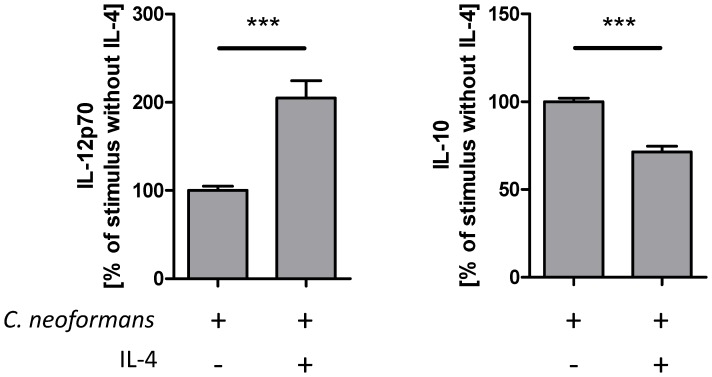
IL-4 induces IL-12 and inhibits IL-10 secretion by conventional dendritic cells stimulated with *C. neoformans*. Conventional BMDCs were generated from bone marrow cells by incubation for 8 to 10 days in the presence of GM-CSF. After harvesting, the cells were stimulated with *C. neoformans* 1841 (MOI 10) in the presence or absence of IL-4 (25 U/ml) for 48 h in duplicates. Supernatants were collected and analyzed for the production of IL-12p70 (n = 3) and IL-10 (n = 4) by sandwich ELISA. The mean of the duplicates without IL-4 was set at 100% and the percentage of each value was calculated in relation to this mean. In a representative experiment 199.5 m±15.5 pg/ml IL-12p70 and 857.5±7.5 pg/ml IL-10 vs. 391.5±2.5 pg/ml IL-12p70 and 696±21 pg/ml IL-10 (mean ± SD) were produced after stimulation with *C. neoformans* in the absence or presence of IL-4, respectively. When incubating cells only in the presence of IL-4 neither IL-12p70 nor IL-10 was detectable (not shown). Data are expressed as the mean ± S.E.M. Statistical analysis was performed by using the unpaired Student's t-test. *** P<0.001.

To characterize the potential mechanism(s) responsible for reduced production of IFN-γ we studied conventional bone marrow derived dendritic cells (BMDC) stimulated with *C. neoformans* in the presence or absence of IL-4. It has been published for experimental leishmaniasis that IL-4 instructs dendritic cells to increase IL-12 production leading to development of Th1 immune responses [18]. Furthermore, it was shown that IL-4 is able to inhibit LPS-induced IL-10 and to enhance IL-12 production by dendritic cells but not by B cells [23]. Thus, we investigated *C. neoformans*-induced secretion of IL-12 by dendritic cells in the presence of IL-4. Indeed, we found that after incubation of conventional BMDC with *C. neoformans* in the presence of IL-4 elevated levels of IL-12p70 are found in supernatants, whereas the production of IL-10 is reduced (Figure 5). In conclusion, besides IL-4Rα-dependent macrophage/dendritic cell recruitment, early IL-4-dependent inhibition of IL-10 may lead to induction of IL-12 by dendritic cells to initiate enhanced IFN-γ production and ultimately allow for better NO-dependent fungal control.

## Discussion

Using a low-dose inoculum of the virulent strain 1841 of *Cryptococcus neoformans* to establish a more chronic course of pulmonary infection we describe for the first time a protective effect of IL-4Rα signaling during the initial phase of the immune response. This observation is in striking contrast to the well-known IL-4Rα-mediated detrimental effects in the advanced state of infection. We conclude that i) IL-4Rα^−/−^ mice are initially more susceptible to infection with *C. neoformans* as shown by stronger fungal growth within the first two weeks of infection. ii) Despite an early type 2 phenotype, WT mice more efficiently control the early fungal growth. IL-4Rα signaling is not only able to enhance the IL-12/IFN-γ/NO axis, but also shows a novel pro-inflammatory activity by up-regulating macrophage and dendritic cell recruiting chemokines such as MCP-1 and MIP-3α.

The unexpected finding of lower fungal lung burdens in WT mice compared to IL-4Rα^−/−^ mice indicates protective effects of IL-4Rα signaling during the onset of immune responses triggered by the infection. This is in accordance with the beneficial effect of IL-4 in *L. major* infection [18]. Furthermore, Yao *et al.* could show an IL-12 inducing effect of IL-4 on LPS-stimulated dendritic cells that is caused by inhibition of IL-10 [23]. In our study the incubation of bone marrow-derived conventional dendritic cells (BMDC) with *C. neoformans* in the presence of IL-4 also resulted in increased IL-12p70 and decreased IL-10 production. Accordingly, we observed reduced IFN-γ expression in the lungs of IL-4Rα-deficient mice at day 14 after infection, pointing to a similar way of action as described for leishmaniasis [18]. When analyzing lung tissue from infected WT mice, we detected a pronounced accumulation of alveolar macrophages and pulmonary dendritic cells as well as large multinuclear macrophages in the lung parenchyma two weeks after infection, suggesting an IFN-γ-dependent influx of antigen-presenting cells [44]. The increased accumulation of alveolar macrophages and dendritic cells in WT mice is accompanied by increased CCL2 and CCL20 mRNA expression in total lung tissue of these mice, pointing to an IL-4Rα-dependent IFN-γ-mediated chemokine production that leads to the attraction of these cells. It was shown previously that IL-12-dependent IFN-γ induces the production of CCL2 and that the lack of CCR2, the receptor for CCL2, abolishes the accumulation of dendritic cells in the lung of *C. neoformans* infected mice [44], [45].

There is substantial evidence that the expression of iNOS and the formation of microbicidial nitric oxide radicals are involved in macrophage-mediated killing of intracellular pathogens such as *C. neoformans* [12], [15], [46], [47]. The IL-4Rα-dependent increased nitric oxide concentration in supernatants from *ex vivo* stimulated lung leukocytes together with the elevated IFN-γ expression in WT mice point to a potential mechanism by which the immune system can reduce the fungal growth in the first two weeks after infection. It was shown that the formation of IFN-γ-induced nitric oxide is necessary to survive a primary infection with *C. neoformans* [48].

Week three after intranasal infection marks a “watershed” for the outcome of pulmonary cryptococcosis – mice which initially control fungal growth lose this ability later on and vice versa (Figure 1, [36]). The regulatory mechanisms that confer susceptibility to WT mice during the third week of infection are presently unclear. One possibility is the potential change in the cell type exerting antigen presentation, i.e. from DCs as early antigen-presenting cells [49], [50] to B cells as later antigen-presenting cells. In the late phase IL-4 may act detrimentally by inducing activation of B cells and isotype switching to IgE [51].

Disease progression in the murine leishmaniasis model is also associated with IL-4 production and development of Th2 immune responses, whereas resistance is mediated by IFN-γ and Th1 cells [52], [53]. During the acute phase of *L. major* infection, IL-4Rα^−/−^ mice can control parasites for the first 80 days. Later on, they show a dramatic progression of disease, whereas IL-4-deficient mice are well protected; pointing to a protective role of IL-13 in leishmaniasis [28]. This is noteworthy and contrary to our pulmonary cryptococcosis model where IL-4Rα^−/−^ mice are completely resistant against *C. neoformans* even over a long period of time (i.e. >200 dpi) [36]. Yet, IL-13 triggers detrimental effects in cryptococcosis [29].

The individual contributions of IL-4 vs IL-13 to induction of type 1 responses remain controversial. It is known that stimulus-induced IL-12 secretion by dendritic cells can be elevated by IL-4R type I signaling reflecting IL-4- (but not IL-13-) dependent Th1 priming by dendritic cells [54]. In addition, IL-13 does not regulate cytokine production by Th1 and Th2 cells in mice [55]. On the other hand it has been shown that both IL-4 and IL-13 are able to promote Th1 immune responses and protection against microbial infections [56]. Recombinant IL-13 increased the production of IL-12 *in vivo* and *in vitro* in a *L. monocytogeneses* infection model, but the early production of IFN-γ was decreased [57]. Webb *et al*. reported also a suppressive effect of IL-13 or an IL-13-dependent factor on IFN-γ production by memory T helper cells in an allergy model [58]. In visceral leishmaniasis both, IL-4 and IL-13 play a positive role in granuloma formation and maturation (also pointing to IL-4Rα-dependent inflammatory cell recruitment as found here for pulmonary cryptococcosis) and are essential for optimal development of IFN-γ responses [59]. In contrast, *in vitro* incubation of BMDC with *Staphylococcus aureus* Cowan I strain in the presence of IL-13 does not influence the production of IL-12, whereas IL-4 increases IL-12 production [54]. Since both, IL-4 and IL-13 bind to IL-4Rα [9], our data from IL-4Rα^−/−^ mice leave to be resolved what the individual contribution of either ligand to early fungal growth control is.

For future studies it would be of great interest to identify the cellular source of early IL-4/IL-13. Our initial data point to the Th cell compartment as the main cellular source of IL-4 and IL-13 early in the infection as revealed by analysis of IL-4 and IL-13 mRNA expression in the enriched pulmonary CD4^+^ population of WT mice (our own unpublished observation). The type 1 immune response-driving potential of IL-4Rα in the early phase of pulmonary infection may be exploited in vaccination strategies against Th2-related pulmonary infection and possibly also in asthma.
